# Role of surgical resection and its alternative local therapy for pulmonary metastasis of colorectal cancer

**DOI:** 10.1002/ags3.12472

**Published:** 2021-05-24

**Authors:** Hiroomi Ogawa, Toshiki Yajima, Makoto Sohda, Ken Shirabe, Hiroshi Saeki

**Affiliations:** ^1^ Department of General Surgical Science Graduate School of Medicine Gunma University Maebashi Japan; ^2^ Department of Innovative Cancer Immunotherapy Graduate School of Medicine Gunma University Maebashi Japan

**Keywords:** colorectal cancer, pulmonary metastasis, pulmonary resection, radiation therapy, radiofrequency ablation

## Abstract

We reviewed surgical and alternative treatments for pulmonary metastasis of colorectal cancer, focusing on recent reports. The standard treatment for pulmonary metastasis of colorectal cancer is pulmonary resection, if resectable, despite the fact that the metastasis is hematogenous to distant organs. Guidelines in several countries, including Japan, have described pulmonary resection as a useful option because of the favorable long‐term prognosis reported in various studies pertaining to pulmonary resection. The indications for pulmonary resection have been reviewed in several studies; additionally, the number of metastases, pretreatment carcinoembryonic antigen value, and disease‐free interval from the primary resection to pulmonary recurrence have been proposed. However, no consensus has been reached to date. Contrastingly, recent advances in chemotherapy have remarkably improved the outcome of distant metastases, indicating that it is time to reconsider the significance of local treatment, including pulmonary resection. In addition to surgical resection, minimally invasive therapies, such as stereotactic body radiation therapy and radiofrequency ablation have been developed as local treatments for pulmonary metastases, and their long‐term results have been reported. Prospective controlled trials and large‐scale data analyses are needed to determine the best local treatment for pulmonary metastases and to find the appropriate indication for each treatment.

## Graphical abstract

In this review, we summarize surgical and alternative treatments for pulmonary metastasis of colorectal cancer, focusing on recent reports. Recent advances in chemotherapy have remarkably improved the outcome of distant metastases, indicating that it is time to reconsider the significance of local treatment, including pulmonary resection. In this era of advanced chemotherapy, the time may no longer allow us to continue to advocate resection as the only curative strategy. It is time for us to build a new strategy for the next era.

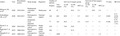

## 1. INTRODUCTION

1

Colorectal cancer is one of the most common cancers in the world, along with breast, pulmonary, and prostate cancers.^1^ In Japan, the number of patients with colorectal cancer is increasing annually. Recently, it has been observed that approximately 153,000 people suffer from colorectal cancer, and approximately 50,000 individuals die due to colorectal cancer each year.^2^, ^3^ Although the relative 5‐y survival rate for colorectal cancer in Japan is approximately 71.4%,^4^ the survival rate varies greatly depending on the stage at the time of diagnosis. Colorectal cancer often metastasizes to the liver and lungs. Pulmonary metastases are the second most common metastatic malignancies following liver metastases.^5^ Metastases have been reported to occur in 19% of patients undergoing radical resection.^6^

There are several reports on the treatment of metastatic colorectal cancer, and the development of chemotherapy in recent years has particularly been remarkable. Over the last decade, treatments for metastatic colorectal cancer have made great strides, with the overall survival improving from 5 mo in 1993 to more than 30 mo.^7^, ^8^

In contrast, there have been several reports on the local treatment for metastatic lesions, and numerous studies on the usefulness of surgical resection have been reported. Accordingly, the main guidelines worldwide now state that the resection of resectable lesions for metastatic colorectal cancer should be considered.^9^, ^10^ Additionally, the Japanese guidelines for the treatment of colorectal cancer state that multidisciplinary treatments, such as systemic chemotherapy, surgical resection, and radiation therapy should be considered.^11^ However, a number of questions remain unanswered, including the usefulness, indications, timing, and order of treatment for resection and other treatments for liver and pulmonary metastases.

Numerous reports have focused on the pulmonary metastasis of colorectal cancer, and several prognostic factors have been proposed. Most reports have examined pulmonary resection; however, almost all reports were retrospective studies, and few were prospectively randomized. Based on these retrospective studies, pulmonary resection has been regarded as the standard treatment for resectable tumors.

Although pulmonary resection is a representative local treatment, radiation therapy and ablation therapy have recently been developed as local treatments to replace pulmonary resection.^12^, ^13^ These have also achieved important results as new treatment options.

This review focuses on the recent reports highlighting the usefulness of surgical resection and alternative treatments as local treatments for pulmonary metastases of colorectal cancer.

## 2. SURGICAL TREATMENT

2

### 2.1 Resection of pulmonary metastases

There has been much debate about the survival benefits of pulmonary resection for pulmonary metastases of colorectal cancer. Although no conclusions have been reached regarding the life‐prolonging effect of pulmonary resection, resection is said to be the only curative treatment; in fact, radical resection for pulmonary metastases has improved the 5‐y survival rate.^14^, ^15^ According to the annual report of the Japanese Association for Thoracic Surgery, resection for metastatic lung tumors was performed in 8950 patients, of whom 4240 patients had pulmonary metastases from colorectal cancer.^16^ According to a report by Murakawa, pulmonary resection for pulmonary metastases of colorectal cancer has doubled every 10 y.^17^

According to the European Society for Medical Oncology (ESMO) guidelines,^9^ systemic therapy is the standard treatment for colon cancer metastasis. Contrastingly, local treatment should be considered as an option only according to the disease site, treatment goal, and patient factors, such as comorbidity and age. The National Comprehensive Cancer Network (NCCN) guidelines^10^ recommend chemotherapy and resection for liver and pulmonary metastases; however, the guidelines recommend chemotherapy and immunotherapy for multiple organ metastases. Although both guidelines have some restrictions on surgery, there are no clear criteria for surgical resection. This is due to the lack of appropriate randomized clinical trials that prospectively considered surgical resection for pulmonary resection. Nevertheless, pulmonary resection is recognized as the standard treatment for resectable pulmonary metastases because some retrospective studies have shown good survival after pulmonary resection. Several retrospective studies have shown an overall 5‐y survival rate of approximately 32.4%–43% after pulmonary metastasis resection,^18^, ^19^, ^20^ and a meta‐analysis reported a 5‐y survival rate of 27%–68%.^21^ In some cases, long‐term survival is considered curative. Unfortunately, pulmonary recurrence can occur after pulmonary resection, for which repeat resection has been reported as a useful treatment. Hishida et al^22^ reported that pulmonary recurrence after resection was found in 24.1% (216/898) of patients, and repeat resection resulted in a 5‐y survival rate of 75.3% in a nationwide survey in Japan. They reported that patients who underwent repeat pulmonary resection had better outcomes than other patients, suggesting that pulmonary resection may be a promising curative treatment at this time. However, Menna et al^23^ reported that there was no significant difference between repeat and single pulmonary resection, suggesting that there are limitations to repeat pulmonary resection. Further studies are needed to determine the patients who benefit from repeat pulmonary resection. In the Japanese guidelines, it was reported that the 5‐y survival rate for pulmonary resection was 46.7% and the cumulative 5‐y relapse‐free survival rate was 33.7%, while the 5‐y survival rate for nonresected cases was 3.9%, based on a multicenter aggregate project study by the Japanese Society for Cancer of the Colon and Rectum (JSCCR).^24^, ^25^ The 5‐y survival rate for pulmonary metastases without resection was considered to be 0%, at least 5% or less in the past.^21^, ^25^ On the other hand, several retrospective studies on pulmonary resection cases have reported the above‐mentioned good treatment results, demonstrating the superiority of resection. There was a small report in 1980 that determined that there was no difference in the survival among 12 patients with resectable pulmonary metastases, who did not undergo resection and 70 patients who did.^26^ However, it was a small‐scale, old study. It is difficult to refer to the results because the medical treatment situation was completely different from that of the present.

A large multicenter prospective clinical trial was planned in Europe to resolve this situation. The Pulmonary Metastasectomy in Colorectal Cancer (PulMiCC) trial was a prospective framework for assigning patients with resectable pulmonary metastases of colorectal cancer to those who underwent metastatic resection and those who did not.^27^ However, the study was discontinued prematurely due to delayed accumulation of cases. Therefore, the originally set statistical analysis has not been performed; however, an update summarizing the results of 93 randomized cases was recently reported.^28^ Notably, the 4‐y survival rate and median survival time of the pulmonary resection group and the nonresection group (control arm) were almost similar (resection group vs. control group = 44% vs. 47%, 3.5 y vs. 3.8 y). Although the study lacked statistical significance, it has a certain clinical value.

No other prospective trials have compared pulmonary resection with nonresection. In contrast, a large retrospective study examining the National Cancer Database (NCDB) has recently been reported.^29^ Of the 600,000 patients with colorectal cancer enrolled in the NCDB, 7217 patients with pulmonary metastases alone were examined, of which 3.63% underwent primary resection, pulmonary resection, and chemotherapy, while 14.7% of the patients were untreated. The overall 5‐y survival rate for pulmonary metastases alone was 16.7%, while the 5‐y survival rate for patients who underwent resection of primary and metastatic lesions and chemotherapy was the best, at 44.52%. However, the prognosis was 24.48% with resection of the primary lesion and chemotherapy, 10.36% with resection of the primary lesion alone, and 9.22% with chemotherapy alone, suggesting that the prognosis was not extremely poor even without pulmonary resection. This indicates that good outcomes are likely to be selected only for patients with limited disease and for a small proportion of patients receiving the treatment.

In another population‐based study, data from the Surveillance, Epidemiology, and End Results Program of the National Cancer Institute in the United States were used to extract 807 patients with simultaneous pulmonary metastases from 217,068 colorectal cancer patients.^30^ Based on the analyses, although pulmonary resection was better for the overall survival and cancer‐specific survival when unadjusted, pulmonary resection did not benefit the overall survival or cancer‐specific survival when adjusted by the propensity score and inverse probability‐weighted adjustment.

Table 1 shows the major recent reports investigating the long‐term survival of patients who underwent pulmonary resection and those who did not.^28^, ^30^, ^31^, ^32^, ^33^ Although the 5‐y overall survival rate of pulmonary resection tends to be better in retrospective studies, the survival rate of nonsurgery is also worthy of comparison.

**TABLE 1:** Major recent reports investigating the long‐term survival of pulmonary resection and nonsurgery

Author	Year	Recruitment period	Study design	Registries	Number of patients		Survival of surgery group			Survival of nonsurgery group			*P*‐value	HR (CI)
					Surgery	Nonsurgery	3y OS (%)	5y OS (%)	MST (y)	3y OS (%)	5y OS (%)	MST (y)		
Milosevic M, et al^28^	2020	2010‐2016	Randomized controlled trial	Multicenter	46	47	—	36.4	3.5	—	29.6	3.8	—	—
Siebenhüner AR, et al^30^	2020	2010‐2015	Retrospective cohort	National registry	144	663	60.0	—	3.5	44.0	—	2.7	.024	0.73 (0.56‐0.97)
			(propensity score adjusted)		77	125	58.9	—	3.4	51.0	—	3.1	.348	0.79 (0.47‐1.31)
Davini F, et al^31^	2020	2010‐2016	Retrospective cohort	Single center	210	—	74.0	54.0		—	—	—	—	—
Rapicetta C, et al^32^	2019	2000‐2016	Retrospective cohort	Single center	344	—		61.9		—	—	—	—	—
Margalit O, et al^33^	2019	2000‐2014	Retrospective cohort	National registry	—	4498	—	—	—	—	—	1.4	—	—

Abbreviations: 3y, 3 year; 5y, 5 year; CI, confidence interval; HR, hazard ratio; MST, median survival time; OS, overall survival.

The prognosis of patients with pulmonary metastases from colorectal cancer has been gradually prolonged in recent reports, regardless of pulmonary resection. This may be due to advances in systemic chemotherapy and improvements in diagnostic imaging techniques, such as the spread of thin‐slice computed tomography (CT) and positron emission tomography (PET), which has resulted in a more accurate diagnosis and reduced oversight of micrometastases.

### 2.2 Prognostic factors and indication for surgery

Metastatic resection of colorectal cancer is not performed in all cases, but in approximately a quarter or a fifth of the cases.^34^ The indications for pulmonary resection were first described by Thomford et al^35^ in 1965. According to the Japanese guidelines,^11^ the criteria for pulmonary resection are: (a) the patient should be capable of tolerating the surgery; (b) the primary tumor has been controlled or can be controlled; (c) the metastatic lung tumor can be completely resected; (d) there are no extrapulmonary metastases or they can be controlled; and (e) the function of the remaining lung will be adequate; however, specific eligibility criteria are not specified. Although numerous studies have been published using the data on survival after surgical resection of pulmonary metastases, identifying the best prognostic factors remains an unsolved problem.

Previous reports have listed high carcinoembryonic antigen (CEA) levels, disease‐free interval (DFI) from primary resection to metastasis, number of metastases, and hilar mediastinal lymph node metastasis as the prognostic factors. A multicenter retrospective study in Japan showed that a DFI of <2 y, presence of extrathoracic lesions, high CEA level, three or more pulmonary metastases, and age >70 y were significantly associated with poor prognosis.^36^ To date, numerous studies have reported that high CEA levels before pulmonary resection correlate with a poor prognosis. However, the cutoff value is not clear, and the CEA value alone is not a criterion as an indication for resection.

Regarding DFI, Yokoyama et al^37^ identified short DFI (<24 mo) and N2 of the primary lesion as risk factors for recurrence after pulmonary resection, but not for survival. Other reports have similarly identified DFI as a risk of recurrence; however, a cutoff for DFI was reported as 36 mo by Davini et al,^31^ while it was determined to be 12 mo by Rapicetta et al.^32^ It is a convincing theory that DFI is a predictor of prognosis, as shorter DFI are expected to be biologically more malignant. However, the cutoff period has not yet been established because there is a great deal of variability in the optimal period.

Several studies have been conducted on the effect of the number of pulmonary metastases on prognosis. Although Pfannschmidt et al^18^ reported that patients with up to four lung metastases showed a significantly better overall survival compared with patients with more than four metastatic lesions, most reported that the patients eligible for pulmonary resection had a single metastasis, based on the results that a single metastasis had a better prognosis.^20^, ^24^, ^38^ On the other hand, Yokoyama et al^37^ pointed out that the number of metastases did not become a prognostic factor, and proposed that the recent advances in thin‐slice CT images and the development of chemotherapy have had a major impact on the results. The same opinion has been proposed in a meta‐analysis by Gonzalez et al,^21^ and with the recent increase in the ability of CT images, we may have to be careful in interpreting the results of previous reports.

The presence of hilar/mediastinal lymph node metastases in several reports has been reported to lead to a poor prognosis.^21^, ^39^ However, some studies have reported that it does not affect the prognosis.^31^ It is considered that the small number of objects has been a limitation in previous analyses, because dissection of the hilar/mediastinal lymph nodes is not a routine procedure in the pulmonary resection for pulmonary metastases. The surgical indications for pulmonary metastasis with hilar/mediastinal lymph node metastases and their effects on the prognosis of lymph node dissection are still controversial.

Regarding the prognosis of patients with pulmonary metastases with a history of hepatectomy, a meta‐analysis and a systematic review in 2013 showed that a history of hepatectomy was not associated with an increased risk of death.^21^ Contrastingly, a subsequent large‐scale meta‐analysis confirmed a history of liver metastases as a poor prognostic factor for pulmonary resection.^40^ However, in the same study the survival rates were comparable between heterogeneous and simultaneous liver and lung metastases. Although these reports are meta‐analyses and have a large number of cases, the interpretation of these results must be carefully performed due to the conspicuous heterogeneity of the patient background. Notably, the history of hepatectomy or the presence of concurrent liver metastases does not appear to be a contraindication for surgery for pulmonary metastases at this time. Additionally, these two meta‐analyses identified other poor prognostic factors. Gonzalez et al^21^ found that short DFI, lymph node infiltration, multiple metastases, and high CEA values were poor prognostic factors. In addition to the above four factors, Zabaleta et al^40^ identified three factors with positive resection margins, large tumor diameters, and a history of liver metastasis as poor prognostic factors.

Davini et al^31^ recently conducted a retrospective cohort study concerning resection margins and reported that shorter margins correlate with poorer prognoses, with distances of 2 cm or more having the best prognosis. Pulmonary‐sparing surgery, such as the wedge resection, is desirable if possible, considering pulmonary recurrence after pulmonary resection. However, since it is a prerequisite to secure a sufficient margin for resection, further discussions including surgical procedures, such as wedge resection and anatomical resection, and approach methods such as open surgery or video‐assisted thoracic surgery are necessary.

### 2.3 Perioperative chemotherapy

The recurrence rates after the resection of pulmonary metastases of colorectal cancer have been reported to be high,^31^, ^41^ and recurrence after pulmonary resection remains a concern. Although the effectiveness of various additional chemotherapies has been investigated to reduce recurrence, no studies have prospectively examined preoperative and postoperative chemotherapy. To date, it is debatable whether perioperative chemotherapy contributes to prolonged survival before and after resection of pulmonary metastases from colorectal cancer.

Several retrospective studies and reviews have reported that perioperative chemotherapy improves the prognosis.^31^, ^42^, ^43^ However, recent meta‐analyses have reached different conclusions. Li and Qin^44^ performed a meta‐analysis of eight studies and found perioperative chemotherapy to be a prognostic factor for favorable overall survival, while Zhang et al^45^ analyzed 18 studies and concluded that postoperative chemotherapy did not provide a significant survival benefit. On the other hand, Rapicetta et al^32^ reported that neither preoperative chemotherapy nor postoperative chemotherapy improved the prognosis, and that survival was slightly worse in those who received chemotherapy than in those who did not, although the difference was not significant. This may reflect the fact that postoperative chemotherapy is administered in high‐risk cases. It is notable that not only do the conclusions vary by study, but all of these are retrospective studies and have different patient backgrounds; thus, these conclusions should be interpreted carefully. In addition, since the regimen is diverse in every report, it is necessary to conduct a well‐controlled prospective study to investigate which drug is effective, for how long, and when to perform it.

## 3. ALTERNATIVE LOCAL THERAPY

3

Stereotactic body radiation therapy (SBRT) and radiofrequency ablation (RFA) have recently attracted attention as local treatments that could replace surgical resection. These therapies meet the standards required for local treatment in terms of the feasibility for patients with poor lung function, lung preservation, and repeatability. A Danish national statistical survey reported an increase in the number of minimally invasive SBRT and RFA treatments for liver and pulmonary metastases over the last 14 y.^46^ Of these, surgical resection demonstrated a higher survival rate than SBRT or RFA, while multivariate analysis showed no significant difference. These treatment selection criteria are not the same, and it is generally considered that RFA/SBRT be selected for patients who are not eligible for surgical resection. No clinical trial has directly compared these three modalities.

### 3.1. Stereotactic body radiation therapy

3.1

SBRT provides an accurate irradiation of lesions and minimal radiation exposure to surrounding normal tissues, making it an alternative treatment option for metastatic resection. SBRT is recommended when surgery cannot be considered, even with the ESMO or NCCN guidelines. In Japanese guidelines, SBRT should be considered when surgery is not tolerated, primary lesions and extrapulmonary metastases are controlled, and the number of metastatic lung tumors is within 5 cm is 3 or less.

Choi et al^12^ conducted a meta‐analysis of 14 studies and examined 495 patients who underwent SBRT for pulmonary metastases. The 5‐y overall survival (OS) rate was 43.0%, the local control rate was 61.8%, and the adverse events of grade 3 or higher were observed in 2.2%. This is comparable to the reports of retrospective trials of pulmonary metastasis resection and the prospective randomized study (PulMiCC) mentioned above. The fact that the results were comparable to resection in this study, which included many cases that were not indicated for pulmonary resection, suggests that SBRT is a candidate for alternative treatment. In contrast, the local control rate after SBRT for pulmonary metastases from colorectal cancer has been reported to be worse than that for pulmonary metastases from other primary sites.^47^ As another treatment option, Takahashi et al^48^ showed that carbon ion radiotherapy (CIRT) could achieve a good local control rate for lung metastases from colorectal cancer without serious side effects, and patients with single lung metastases from colorectal cancer are the best candidates for CIRT.

However, the average number of metastases per patient treated with SBRT and CIRT was 1.4 and 1, respectively, and the therapeutic effect of multiple lesions was unknown. As mentioned above, assuming that the number of pulmonary metastases suitable for pulmonary resection is one, SBRT and CIRT are not only an alternative treatment but also a sufficiently powerful treatment option. However, the treatment targets of these therapies are still limited.

### 3.2. Radiofrequency ablation

3.2

RFA has been widely used in the last decade as a local treatment alternative to surgical treatment. In an international study in 2004, RFA was reported as a useful minimally invasive tool for local treatment with low mortality and complications. Long‐term survival for RFA treatment has also been reported, with a median OS of 52 mo in a recent report, compared to 33–50 mo in the past.^49^, ^50^ A systematic review of 903 patients in eight studies summarized that RFA is a safe and effective treatment for pulmonary metastases of colorectal cancer, with 5‐y survival rates ranging from 20%–54% and serious complications from 0.5%–8%.^13^

Since RFA is less invasive than surgical resection and is advantageous for lung preservation, it is considered a suitable treatment for cases in which surgery is not indicated or repeated treatment is expected. However, Hiyoshi et al^49^ reported that ≥15 mm is an independent poor prognostic factor, which limits its indications as an alternative to surgical treatment. In addition, most retrospective studies are mixed cohorts of surgical resection and other local treatments and systemic chemotherapy, and there have been insufficient analyses of the oncologic prognosis of RFA alone. Therefore, several authors have stated that prospective randomized controlled trials are required.

Recently, alternatives to RFA, such as microwave ablation and cryoablation, have been developed and reported to be effective in local control; however, there is not yet enough evidence for them to be useful in clinical practice.^51^, ^52^

## 4. COMMENTS

4

In the past, resection was considered the only effective treatment for pulmonary metastases; in fact, some guidelines such as NCCN and ESMO also recommend resection. The Japanese guidelines also weakly recommend resection. However, numerous reports in recent years have cast doubt on this stereotype.

SBRT is beginning to show results that are less toxic to patients in poor condition and leads to long‐term survival. Although RFA is restricted in its indications due to tumor size and other factors, it offers the advantage of local control, such as the ability to perform repeated treatments. Resection, SBRT, and RFA are still local treatments for pulmonary metastases. From that point of view, it is understandable that these outcomes are similar.

Recent chemotherapy has made rapid progress, and the development and progress of various chemotherapies have made it possible to significantly extend survival. Currently, several patients achieved long‐term survival without surgery. If distant metastasis is considered a systemic disease, the underlying treatment is systemic therapy, that is, chemotherapy. Given that these local therapies act on some of the diseases, it is evident that chemotherapy is a major factor in determining the ultimate survival. The PulMiCC trial is a notable suggestion, albeit a discontinued trial.

Although the survival benefits of pulmonary resection for pulmonary metastases from colorectal cancer are controversial, it is true that some patients will benefit from long‐term survival or cure from pulmonary resection of pulmonary metastases. Local treatment needs to be sought for its potential to be an extremely effective tool for a more optimized patient population.

Well‐controlled prospective trials that directly compare resection with these treatments are not yet sufficient. However, in this era of advanced chemotherapy, the time may no longer allow us to continue to advocate resection as the only curative strategy. It is time for us to build a new strategy for the next era.

## DISCLOSURE

Conflict of Interest: The authors have no potential conflicts of interest to disclose.
